# Variability in HIV-1 Integrase Gene and 3′-Polypurine Tract Sequences in Cameroon Clinical Isolates, and Implications for Integrase Inhibitors Efficacy

**DOI:** 10.3390/ijms21051553

**Published:** 2020-02-25

**Authors:** Arpan Acharya, Claude T. Tagny, Dora Mbanya, Julius Y. Fonsah, Emilienne Nchindap, Léopoldine Kenmogne, Ma Jihyun, Alfred K. Njamnshi, Georgette D. Kanmogne

**Affiliations:** 1 Department of Pharmacology and Experimental Neuroscience, College of Medicine, University of Nebraska Medical Center, Omaha, NE 68198, USA; arpan.acharya@unmc.edu; 2 Faculty of Medicine and Biomedical Sciences, University of Yaoundé I, P.O. Box 1364 Yaoundé, Cameroondmbanya1@yahoo.co.uk (D.M.); funsah@yahoo.co.uk (J.Y.F.); alfredknjamnshi@gmail.com (A.K.N.); 3 Yaoundé University Teaching Hospital, Department of Haematology, P.O. Box 5739 Yaoundé, Cameroon; eminchindap@yahoo.com (E.N.); l.kemmogne@yahoo.com (L.K.); 4 Department of Neurology, Yaoundé Central Hospital/Brain Research Africa Initiative (BRAIN), P.O. Box 25625 Yaoundé, Cameroon; 5 Department of Biostatistics, College of Public Health, University of Nebraska Medical Center, Omaha, NE 68198, USA; jihyun.ma@unmc.edu

**Keywords:** HIV, integrase inhibitors, 3′polypurine tract, antiretroviral drugs, resistance-associated mutations, Cameroon

## Abstract

Integrase strand-transfer inhibitors (INSTIs) are now included in preferred first-line antiretroviral therapy (ART) for HIV-infected adults. Studies of Western clade-B HIV-1 show increased resistance to INSTIs following mutations in integrase and nef 3′polypurine tract (3′-PPT). With anticipated shifts in Africa (where 25.6-million HIV-infected people resides) to INSTIs-based ART, it is critical to monitor patients in African countries for resistance-associated mutations (RAMs) affecting INSTIs efficacy. We analyzed HIV-1 integrase and 3′-PPT sequences in 345 clinical samples from INSTIs-naïve HIV-infected Cameroonians for polymorphisms and RAMs that affect INSTIs. Phylogeny showed high genetic diversity, with the predominance of HIV-1 CRF02_AG. Major INSTIs RAMs T66A and N155K were found in two (0.6%) samples. Integrase polymorphic and accessory RAMs found included T97A, E157Q, A128T, M50I, S119R, L74M, L74I, S230N, and E138D (0.3′23.5% of samples). Ten (3.2%) samples had both I72V+L74M, L74M+T97A, or I72V+T97A mutations; thirty-one (9.8%) had 3′-PPT mutations. The low frequency of major INSTIs RAMs shows that INSTIs-based ART can be successfully used in Cameroon. Several samples had ≥1 INSTIs accessory RAMs known to reduce INSTIs efficacy; thus, INSTIs-based ART would require genetic surveillance. The 3′-PPT mutations could also affect INSTIs. For patients failing INSTIs-based ART with no INSTIs RAMs, monitoring 3′-PPT sequences could reveal treatment failure etiology.

## 1. Introduction

During HIV replication, the integrase enzyme catalyzes the ligation of viral reverse-transcribed DNA into the chromosome of the host cells. This integration is a critical step for viral replication, enabling the proviral DNA to persist in the host cells and form a permanent viral template that can continuously initiate the production of new viruses and replicate with each cell division [1,2,3]. Integrase is a 32 kDa protein (288 amino acids (aa)) encoded by the C-terminal of HIV polymerase gene [4]. It has 3 functional domains: an N-terminal domain (aa 1-49) that facilitates protein multimerization; a catalytic core domain (aa 50-212) that contains the viral DNA binding site and is involved in the recognition of viral DNA substrate; and a C-terminal domain (aa 213-288) that help stabilize the integrase–viral DNA complex [4,5,6].

Considering its critical role in viral integration into the host genome and the formation of viral reservoirs, integrase has been a major HIV/AIDS therapeutic target. There are currently three FDA-approved integrase strand transfer inhibitors (INSTIs) used as part of antiretroviral therapy (ART) regimens [raltegravir (RAL), elvitegravir (EVG), and dolutegravir (DTG)], and a fourth INSTI, bictegravir, approved for use as a component of a fixed-dose combination ART [7]. Compared to other drug classes, these INSTIs have shown higher efficacy, superiority and higher genetic barrier to the development of resistance [8,9]. Thus, INSTIs are now recommended as part of initial or preferred ART regimens for most HIV-infected people in western countries [7,10] and are also recommended by the World Health Organization (WHO) as alternative 1st-line regimens for adults living with HIV/AIDS [11,12]. However, access to INSTIs in resource-limited countries has been restricted due to its high costs. Although WHO is now recommending DTG as alternative 1st-line regimen [11,12], its use is currently rare in resource-limited countries and it is estimated that only two countries in Sub-Saharan Africa (SSA), Kenya and Botswana, are currently providing DTG to HIV-infected persons as part of 1st-line ART regimens [13,14]. Nevertheless, with DTG license to generic pharmaceutical manufacturers through the Medicines Patent Pool [15], and recently negotiated reduced DTG prices through collective bargaining with many countries [15,16,17], INSTIs are expected to be increasingly available in several resource-limited countries. In fact, although global 2018 estimates show that DTG was used in only 4% of 1st-line ART regimens and in 6% of 2nd-line regimens, it is projected that by the year 2025, DTG, including single pills containing DTG and other antiretroviral drugs (ARVs), will be used by up to 57% of people living with HIV (PLWH) [16]. 

There is evidence of a worldwide increase in HIV resistance to many ARVs, including non-nucleoside reverse transcriptase inhibitors that form the backbone of many current WHO-recommended 1st- and 2nd-line regimens [11,12]; and many people failing these 1st- and 2nd-line regimens will require 3rd-line regimens and INSTIs as rescue therapies [11,12,18]. Resistance to ARVs is associated with mutations in the viral genome, including transmitted and acquired drug-resistance mutations (DRMs) [19]. These DRMs are associated with increased risk of treatment discontinuation, virological failure, and death [19]. It has also been shown that people with pretreatment DRMs are more likely to develop new DRMs after initiating treatment [19].

Despite their higher efficacy, evidence from HIV-infected people on INSTIs in western countries showed resistance to INSTIs, including DRMs resulting in treatment failures [20,21,22,23], and polymorphic mutations that contribute to the rescue of viral fitness and increased resistance to INSTIs [22,23]. All these evidences are from populations in developed countries, and there are major knowledge gaps on INSTIs resistance-associated mutations (RAMs) across diverse populations, especially in Sub-Saharan Africa (SSA) where over two-thirds of the current 38 million PLWH reside [24]. To address this scientific knowledge gap, WHO recently recommended that HIV drug resistance surveillance, including pretreatment DRMs, be implemented for INSTIs [18,19]. These recommendations are critically important considering the current shift of many ART programs to INSTIs-based regimens [7,10] and anticipated shifts in SSA and other resources-limited countries [16]. Therefore, the goal of our current study was to analyze integrase gene sequences in clinical samples to assess the occurrence of polymorphisms and INSTIs RAMs in PLWH in Cameroon, a SSA country that has not yet adopted INSTIs-based ART. Because it has been shown that mutations located outside the integrase gene, in the viral 3′ polypurine tract (PPT, located in the 3′ end of nef), can confer resistance to INSTIs [25,26,27], we also sequenced and analyzed nef and the PPT viral genome region in these samples. We further analyzed the HIV integrase gene and PPT sequences from 215 additional patient samples from Cameroon, obtained from the Los Alamos database [28], for INSTIs RAMs and natural polymorphisms that could affect INSTIs-based therapy.

## 2. Results

### 2.1. Patient Demographics

We collected samples from 235 HIV-infected subjects in Cameroon, of which we successfully amplified and sequenced the integrase and/or nef genes in 130 samples (Table 1). This cohort of 130 patients included 39 males [mean age: 37.28 ± 9.94 years] and 91 females (35.43 ± 8.75 years). The mean plasma viral load and CD4+ T-cells count at the time of specimen collection were, respectively, 4.64 ± 1.54 log copies/mL and 360.3 ± 320.4 cells /µL for males and 4.24 ± 1.50 log copies/mL and 311.2 ± 184.5 cells /µL for females. Of the samples with successfully amplified and sequenced integrase and nef genes, 33 (84.61%) males and 66 (72.52%) females were treatment-naïve, 5 (12.82%) males and 25 (27.47%) females were on ART (1st-line regimens), 1 female had discontinued treatment, and treatment information was not available for 1 male. None of the cohort patients had been exposed to INSTIs at the time of enrolment and specimen collection. We also analyzed 215 full-length Cameroon HIV-1 sequences (samples collected from HIV+ Cameroonians between 1991 and 2014, when INSTIs was not available in Cameroon and many other countries) previously deposited in the Los Alamos HIV sequence database (see Table S1). Most of the database sequences information did not include demographics, treatment status, CD4 levels, or viral loads data. 

### 2.2. HIV-1 Subtyping

**Figure 1:**
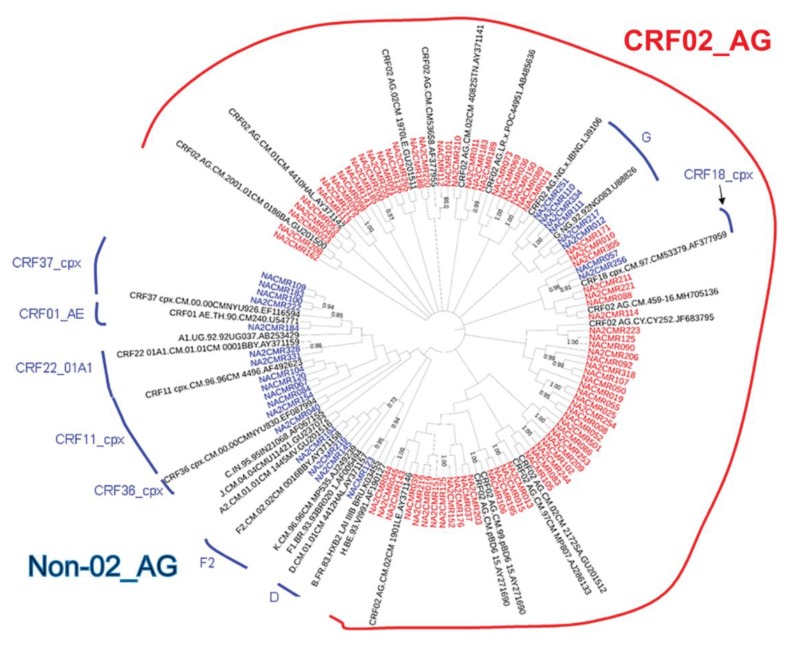
Phylogenetic analysis of HIV-1 integrase gene sequences in cohort samples. The maximum likelihood phylogenetic tree was constructed using the MEGA 6 software package as described in Materials and Methods. The 31 reference sequences were from the Los Alamos database and included HIV-1 isolates from ten different countries [Belgium (1), Brazil (1), Cameroon (21), Cyprus (1), India (1), France (1), Liberia (1), Nigeria (2), Uganda (1), and United Arab Emirates(1)]. Bootstrap values of 1000 replicates (>70%) are shown at the corresponding nodes.

**Figure 2:**
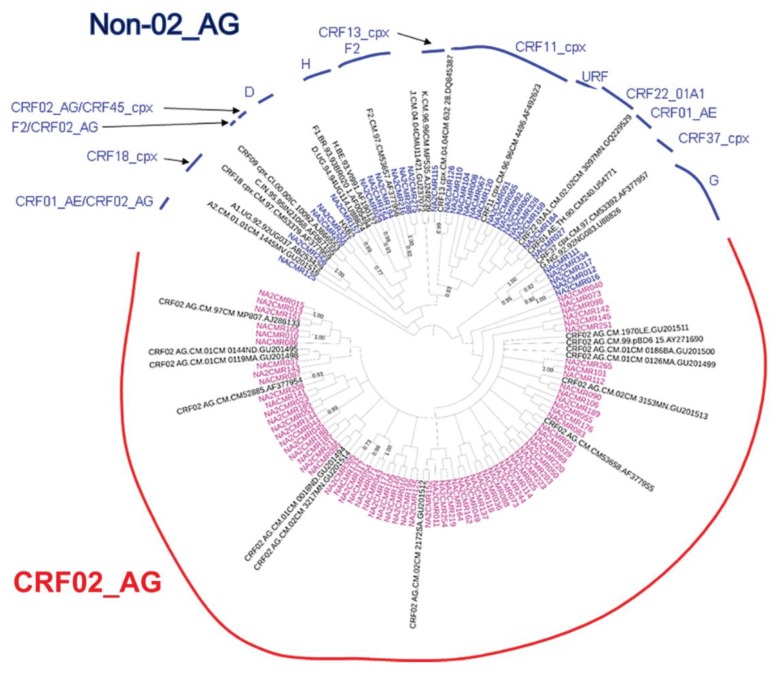
Phylogenetic analysis of HIV-1 nef gene sequences in cohort samples. The maximum likelihood phylogenetic tree was constructed using the MEGA 6 software package as described in Materials and Methods. The 31 reference sequences were from the Los Alamos database and included HIV-1 isolates from nine different countries [Belgium (1), Brazil (1), Cameroon (22), Cote D’Ivoire (1), India (1), France (1), Nigeria (1), Uganda (2), and United Arab Emirates(1)]. Bootstrap values of 1000 replicates (>70%) are shown at the corresponding nodes.

**Figure 3:**
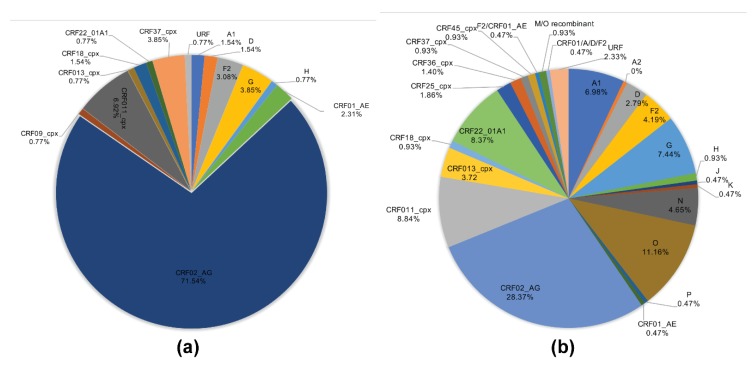
HIV-1 genetic diversity in cohort and database samples. (**a**) Proportions of each HIV-1 subtype and CRFs identified in cohort samples based on NCBI viral genotyping tool (combined analysis of integrase and nef gene sequences). (**b**) Proportions of each HIV-1 subtype and CRFs identified using full-length sequence of database samples, based on NCBI viral genotyping tool. CRF: circulating recombinant form; URF: unique recombinant form.

The viral subtypes of our study cohort were determined using the NCBI viral genotyping tool and phylogenetic analyses as described in Materials and Methods. Of the 100 samples successfully sequenced for the integrase gene, 75% were HIV-1 CRF02_AG (AG) and 25% were non-CRF02_AG (non-AG), including 6% subtype G, 4% each for CRF37_cpx and CRF11_cpx, 3% subtype F2, 2% each for CRF18_cpx and CRF22_01A1, and 1% each for subtype A1, D, CRF01_AE, CRF36_cpx (Figure 1). Of the 101 samples successfully sequenced for the nef gene, 69.31% were HIV-1 CRF02_AG and 30.69% were non-AG subtypes, including 8% CRF11_cpx, 5% subtype G, 4% subtype F2, 3% CRF22_01A1, 2% subtype H; 1% each for subtypes D, CRF01_AE, CRF13_cpx, CRF18_cpx, CRF37_cpx, CRF01_AE/CRF02_AG, CRF02_AG/CRF45_cpx, F2/CRF02_AG, and 1% unique recombinant form (URF) (Figure 2). Combined analyses based on both integrase and nef genes showed that 71.54% of samples were CRF02_AG and 28.46% were non-AG: 6.92% CRF11_cpx, 3.85% each subtypes G and CRF37_cpx, 3.08% subtype F2, 2.31% CRF01_AE, 1.54% each subtypes A1, D, and CRF18_cpx, 0.77% each subtypes H, CRF09_cpx, CRF13_cpx, CRF22_01A1, and a URF (0.77%) (Figure 3a).

Genotypes for the 215 database HIV samples as reported in their original sequences submissions included 180 (83.72%) group M, 24 (11.16%) group O, 10 (4.65%) group N, and 1 (0.47%) group P. The 180 HIV-1 group M samples included 61 (28.37%) CRF02_AG, 19 (8.84%) CRF09_cpx, 18 (8.37%) CRF22_01A1, 16 (7.44%) subtype G, 15 (6.98%) subtype A1, 9 (4.19%) subtype F2, 8 (3.72%) CRF13_cpx, 6 (2.79%) subtype D, 5 (2.33%) URFs, 4 (1.86%) CRF25_cpx, 3 (1.4%) CRF36_cpx, 2 (0.93%) each for subtypes H, CRF18_cpx, CRF37_cpx, CRF45_cpx, and M/O recombinant; 1 (0.47%) each for subtypes A2, J, K, CRF01_AE, F2/CRF02_AG, CRF01_AE/A/D/F2 (Figure 3b). 

### 2.3. INSTI Resistance Mutations in Cohort Samples

No INSTIs major RAMs were present in our cohort samples. The INSTIs accessory RAMs E157Q and T97A were present, respectively, in 6 (all CRF02_AG) and 2 (1 CRF02_AG and 1 CRF09_cpx) samples (Table 2). Four other mutations were identified in cohort samples: M50I (in 6 samples), S119R (in 4 samples, all CRF02_AG), L74M (in 8 CRF02_AG and 1 CRF11_cpx samples), and L74I in 25 samples of which 22 (88%) were CRF02_AG (Table 2). 

### 2.4. INSTI Resistance Mutations in Database Samples

Two INSTIs major RAMs were identified in two database samples: T66A in 1 (0.47%) HIV-1 group M/O recombinant sample isolated in 1997 from a 29 years old ART-naïve Cameroonian female (GenBank accession number: AJ239083) [29]; and N155K in 1 (0.47%) CRF36_cpx sample isolated in 2000 from a 19 years old female in Cameroon (GenBank accession number: EF087995) [30] (Table 3). Three INSTIs accessory RAMs were identified: T97A (in 16 samples), A128T (in 1 M/O recombinant sample), and E157Q (in 3 samples) (Table 3). Other mutations in database samples included M50I, L74M, L74I, S119R, S230N, and E138D, identified in 67 (31%), 9 (4%), 49 (22.8%), 1 (0.47%), 2 (0.93%), and 1 (0.47%) of database samples (Table 3), respectively. 

### 2.5. INSTIs Resistance-Associated Mutations in Subjects Infected with HIV-1 CRF02_AG and Non-AG Viruses

The INSTIs accessory RAMs identified in both cohort and database samples included T97A, E157Q, M50I, L74M, L74I, and S119R. The INSTIs accessory RAMs identified in both HIV-1 CRF02_AG and non-AG cohort samples included T97A, M50I, L74M, and L74I (in 1.3% to 29.3% of AG and in 4% to 16% of non-AG cohort samples). E157Q and S119R mutations were observed only in cohort samples of AG subtype. There were no significant differences in the proportions of AG and non-AG cohort samples with INSTIs RAMs (Table 4). 

The INSTIs accessory RAMs identified in both AG and AG database samples included T97A, E157Q, M50I, L74M, L74I, and S230N (in 3.5% to 60% of AG and in 1.4% to 86.5% of non-AG database samples). A significantly higher proportion of non-AG database samples (86.56%) had the M50I mutation compared to 17.6% in database AG samples (*p* < 0.000001, Table 4). A significantly higher proportion of AG database samples (21%) had the L74M mutation compared to 4.2% in database non-AG samples (*p* < 0.005, Table 4). The proportion of AG and non-AG database samples harboring other INSTIs RAMs was not significantly different and T66A, N155K, A128T, S119R, and S230N were observed only in a very small number (1.4%) of database samples of non-AG genotypes (Table 4). 

### 2.6. Integrase Natural Polymorphisms in Subjects Infected with HIV-1 CRF02_AG and Non-AG Viruses

Similar polymorphisms (substitutions at similar aa positions) were observed in cohort and database samples, including a total of 27 natural polymorphisms (Table 5). 

Integrase polymorphisms in cohort samples: Comparative analyses of cohort subjects infected with CRF02_AG and non-AG subtypes showed that the G134N, I135V, K136T, and T206S polymorphisms were present in a significantly higher proportion of samples with AG subtype (81% to 97%) compared to samples with non-AG subtypes (28–52%) (*p* < 0.00001, Table 5). Similarly, T124A was present in a higher proportion of AG (90.67%) than non-AG (68%) samples (*p* = 0.031, Table 5), and R269K was only present in AG (30.67%) samples (*p* = 0.003, Table 5). For the cohort group, polymorphisms A21T, I72V, D167E, and D256E were significantly more prevalent in non-AG (24–48%) than in AG (5–20%) samples (*p* < 0.04, Table 5) and K136Q was only present in non-AG (44%) samples (*p* < 0.000001, Table 5).

Integrase polymorphisms in database samples: The L101I, T125A, G134N, I135V, K136T, T206S, T218I, and R269K polymorphisms were present in a significantly higher proportion of AG (44% to 95%) compared to non-AG (5–66%) database samples (*p* < 0.00002, Table 5). Similarly, K14R, V31I, T112V, and T214A polymorphisms were present in a significantly higher proportion of AG (75% to 95%) than non-AG (50% to 78%) database samples (Table 5). The E11D, A21T, G134D, K136Q, D167E, I208L, and D256E polymorphisms were significantly more prevalent in non-AG (14′51.3%) than in AG (3–26%) database samples (Table 5).

Integrase polymorphisms in both cohort and database samples: Overall, T124A, G134N, I135V, K136T, T206S, and R269K polymorphisms were significantly more prevalent in AG compared to non-AG samples, whereas A21T, K136Q, D167E, and D256E polymorphisms were significantly more prevalent in non-AG compared to AG samples. 

### 2.7. Effects of ART and Immune Function on Integrase RAMs and Natural Polymorphisms

Additional analyses of cohort patients showed no significant differences in the occurrence of gene polymorphism or gene mutations among subjects who were treatment-naïve and those on ART. Similarly, there were no significant differences in the occurrence of gene polymorphism or INSTIs RAMs in cohort patients with CD4+ T-cell counts above 200 cells/μL (66%) and those with CD4+ T-cell counts below 200 cells/μL (34%). Integrase sequences for Cameroon HIV isolates downloaded from the Los Alamos database did not have information on patient’s treatment status or levels of CD4+ cells.

### 2.8. Analysis of 3′-PPT and 5′ Terminal Nucleotides of 3′ Long Terminal Repeat

**Figure 4:**
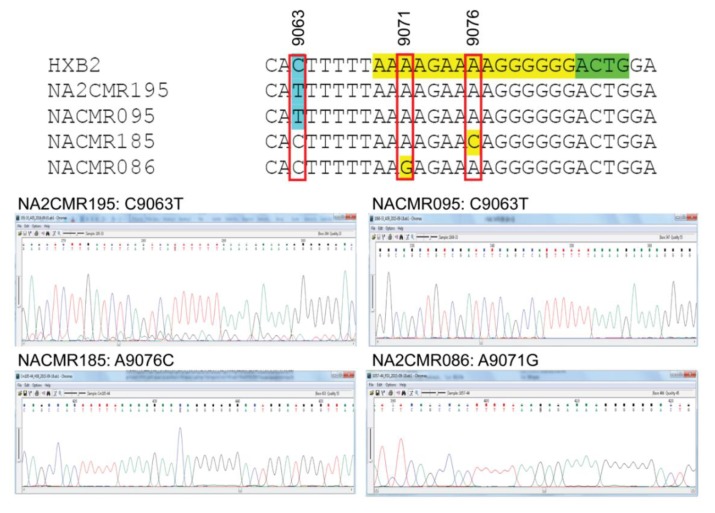
Mutations detected in the 3′polypurine tract (3′-PPT) and its flanking regions for cohort samples. Nucleotide sequences alignments, using HXB2 as a reference sequence, are shown for the four study cohort samples in which mutations were detected. The 15 nucleotides of 3′-PPT sequences are shaded yellow. The 1st four nucleotides of 3′LTR are shaded green. Region with mutation upstream of the 3-PPT (nucleotide position 9063) is shaded in sky blue. Nucleotide numbers correspond to the numbering of the HXB2 reference sequence. Electropherograms showing data sequences and mutations are shown for the four cohort samples.

Because mutations in the HIV-1 nef /3′-PPT region can confer resistance to INSTIs [25,26,27], we also analyzed the sequences of the 3′-PPT viral genome, as well as the 8 nucleotides upstream of the 3′-PPT region (of nef), and the first four nucleotides downstream of the 3′-PPT [5′ terminal region of the 3′ long terminal repeat (LTR)], in comparison to the reference HXB2 genome sequence. Mutations within the 15 nucleotides 3′-PPT region (Figure 4, yellow highlight) was observed only in 2 cohort samples: A9076C mutation in subject NACMR185 and A9071G mutation in subject NACMR086 (Figure 4). Two other cohort samples (NA2CMR195 and NACMR095) had a C to T substitutions in the nef region upstream of the 3′-PPT region (nucleotide 9063). No mutation was observed downstream of the 3′-PPT (LTR) among cohort samples. 

**Figure 5:**
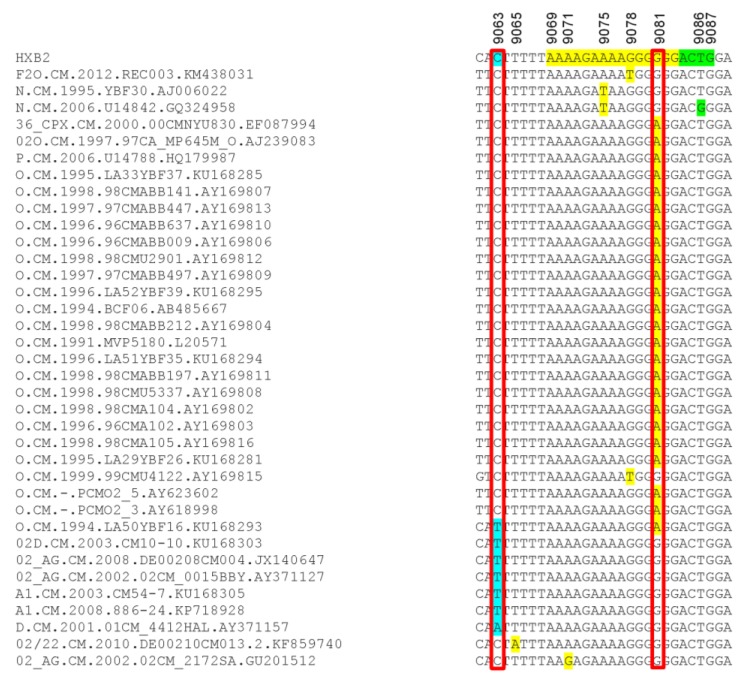
Mutations detected in the 3′-PPT and its flanking regions for database samples. The nucleotides alignment, using HXB2 as a reference sequence, are shown for the database samples in which mutations were detected. The 15 nucleotides of 3′-PPT sequences are shaded yellow. The 1st four nucleotides of 3′LTR are shaded green. Region with mutation upstream of the 3-PPT (nucleotide position 9063) is shaded in sky blue. Nucleotide numbers correspond to the numbering of the HXB2 reference sequence.

Analysis of the 215 database samples showed 29 samples with mutations in the viral 3′-PPT region [G9078T in one subtype F2 and one Group O; A9075T in two HIV-1 group N samples; G9081A in one HIV-1 CRF_36cpx, one HIV-1 group M/O recombinant, one HIV-1 group P, and 21 HIV-1 group O; A9071G mutation in one HIV-1 CRF02_AG sample] (Figure 5). Seven database samples had mutations upstream of the 3′-PPT region (C9063T in one Group O and 5 group M [3 CRF02_AG and 2 subtypes A1]; C9063A in one subtype D) (Figure 5). Mutation downstream of the 3′-PPT (LTR) was only observed in one database sample (T9086G in one Group N sample) (Figure 5).

## 3. Discussion

Despite INSTIs’ high efficacy and superiority compared to other ARV drug classes, studies in high-income countries show that resistance to INSTIs can occur, associated with transmitted and/or acquired DRMs, leading to decreased susceptibility to INSTIs and treatment failure [20,21]. The integrase mutations often associated with reduced susceptibility to INSTIs include both nonpolymorphic and polymorphic mutations [1,22,23]. In 2018, ART regimens including 2nd-generation INSTIs such as DTG constituted only 4% of 1st-line regimens and 6% of 2nd-line regimens worldwide [12,16]. However, INSTIs are now part of the WHO-recommended alternative 1st-line regimens for PLWH [7,11,12]. With ongoing efforts to expand INSTIs availability worldwide, it is projected that by the year 2025, up to 57% of PLWH will be on ART regimens containing DTG or other INSTIs [16]. Therefore, it is important to monitor PLWH for the presence of mutations in the integrase gene that can affect INSTIs efficacy and therapeutic outcomes. Furthermore, our current knowledge of INSTIs RAMs mostly comes from studies in western countries; patient-derived data on INSTIs RAMs in SSA are scarce and there is a major knowledge gap on INSTIs RAMs in SSA, whereas over two-thirds of the current 38 million PLWH reside in that region. Our current study contributes to filling that gap and addresses recent WHO recommendations that HIV drug resistance surveillance be implemented for INSTIs, including pretreatment DRMs [18,19]. We analyzed integrase gene sequences from Cameroon clinical isolates for the presence of mutations known to be associated with resistance or reduced susceptibility to INSTIs. Because mutations in the nef 3′-PPT region have been associated with resistance to INSTIs [25,26,27], we also analyzed nef gene sequences, their 3′-PPT regions, and sequences adjacent to the 3′-PPT regions. 

Phylogeny of integrase and nef sequences showed high genetic diversity, with a predominance of AG infections (69–75% of cohort samples). This is similar to previous studies showing that over 60% of HIV+ Cameroonians harbored AG viruses [31,32,33]. The lower proportion of AG infections seen in database samples (28%) is likely due to overrepresentation of other genotypes such as HIV-1 groups O, N, and P from Cameroon in the database, from studies that specifically analyzed sequences from these subtypes [34], rather than sequences from randomly collected patient samples as in our cohort or other studies of Cameroon isolates [31,32,33]. 

No INSTIs major RAMs were present in cohort samples, and only 2 database samples had INSTIs major RAMs: T66A in a group M/O recombinant and N155K in a CRF36_cpx sample. Mutations in integrase aa 66 can significantly reduce viral susceptibility to INSTIs. T66A and T66I reduce EVG susceptibility, respectively, by 5-fold and 10-fold [35,36,37]. T66K reduced susceptibility to EVG by 40-fold, reduced susceptibility to RAL by 10-fold, and reduced susceptibility to DTG by 2- to 3-fold [21,36,38]. Mutations in integrase aa 155 have been associated with reduced susceptibility to RAL (by 10-fold) and EVG (by 30-fold) [35,39,40], and studies of patients failing DTG-based ART showed that patients with virologic failure had N155H substitution at baseline and also developed additional INSTIs-RAMs [41,42]. The fact that only 0.6% of samples analyzed had INSTIs major RAMs suggests that there will be a lesser likelihood of resistance to INSTIs in Cameroon and that INSTIs will be effective for HIV-infected Cameroonians. Similar trends were observed in other countries in Africa [13,34,43,44,45], Asia [46,47,48,49,50,51], Europe [52,53,54], and South America [55] where studies showed a low frequency of INSTIs major RAMs.

Several other integrase accessory RAMs and polymorphic mutations were observed in our current study, including the INSTIs accessory RAMs T97A (in 2% of cohort and 7.4% of database samples), E157Q (in 6% of cohort and 1.4% of database samples), A128T (in 0.47% of database samples); the polymorphic mutations M50I (in 6% of cohort and 31% of database samples), S119R (in 4% of cohort and 0.47% of database samples), L74M (in 9% of cohort and 4% of database samples), L74I (in 25% of cohort and 23% of database samples), E138D (in 0.47% database samples), and S230N (in 0.93% of database samples). Ten of the 11 INSTIs accessory RAMs observed were in the integrase central core domain. This integrase region contains the endonuclease and polynucleotidyl transferase site and is involved in DNA substrate recognition, binding, and chromosomal integration of the newly synthesized double-strand viral DNA into the host genomic DNA [56,57,58]. The other mutation (S230N) was in the C-terminal domain, a region that helps stabilize the integrase–viral DNA complex [4,5,6]. The location of these mutations in regions involved in recognition, binding, integration, and stabilization of HIV into the host DNA shows the potential for these mutations to affect the integrase function and response to INSTIs. In fact, mutations in integrase aa residue 230 reduce DTG susceptibility by 3-fold [59]. T97A mutation can reduce EVG susceptibility by 3-fold [36], and combination of T97A substitution with other INSTIs RAMs markedly reduce HIV susceptibility to RAL [60,61] and DTG [62,63]. Similarly, although L74M and E157Q individually have minimal effect on the susceptibility to INSTIs, a combination of L74M and E157Q with other INSTIs RAMs result in reduced susceptibility to INSTIs. In fact, patients harboring viruses with L74M [61] or E157Q [64] mutations in addition to other integrase RAMs showed reduced susceptibility to DTG. 

Six of our subjects (3 cohort and 3 database samples) were infected with viruses that had both I72V and L74M mutations; one cohort sample had both L74M and T97A, and 3 database samples had both I72V and T97A mutations. These subjects could be susceptible to resistance to INSTIs, as there is evidence that the presence of multiple integrase accessory RAMs or polymorphic mutations can increase viral fitness and reduce susceptibility to INSTIs, even in the absence of INSTIs major RAMs [40,65]. Furthermore, the fact that most of the integrase mutations observed in our study occurred in the central core and C-terminal domains suggests that they could alter proviral DNA binding, integration, and stabilization and affect INSTIs efficacy. Thus, vigilance and surveillance for INSTIs DRMs would be required in Cameroon and other SSA countries when they do shift to current WHO-recommended DTG/INSTIs-based ART.

Previous studies showed that polymorphic differences among HIV subtypes can affect viral fitness and resistance pathways [66] and that different viral subtypes may support different mutational pathways and this could result in subtype-based differences in drug resistance [67,68,69]. There is also evidence that natural polymorphisms associated with integrase activity and occurrence of resistance to INSTIs are subtype-dependent and subtype-specific polymorphic mutations in the integrase gene affect integrase DNA binding affinity when additional mutations are present and can influence INSTIs efficacy [66,68,70,71,72]. Thus, we compared the occurrence of INSTIs RAMs among samples from subjects infected with HIV-1 CRF02_AG and subjects harboring non-AG subtypes. Our data showed polymorphisms and mutations in the integrase in both groups, similar to previous studies showing that mutations and resistance to INSTIs can occur in both clades-B, CRF02_AG and non-AG subtypes [70,71,73]. However, the frequencies of such mutations can vary based on viral genotype [74]. The aa substitutions E92Q, S119R, E138A, Y143R, G148H/R, and S230R/N are more prevalent in subtype-B than in non-B subtypes [71,74,75,76], whereas mutations such as L74I/M, T97A, L101I, E157Q, T214A, and V201I are more prevalent in non-B subtypes compared to HIV-1 subtype B [71,74,77]. Because the vast majority of PLWH on INSTIs are people infected with HIV-1 subtype B, most INSTIs resistance-conferring mutations that have been characterized pertain to HIV-1 subtype B. It is likely that several mutations prevalent in non-B subtypes could affect the genetic barrier and INSTIs efficacy. In fact, computational modeling of INSTIs resistance development across different HIV-1 subtypes showed that compared to subtype B, the presence of M50I in subtypes A and C, L74I in subtypes A and CRF02_AG, G163R in CRF01_AE, and V165I in subtypes F and CRF01_AE would be associated with lower genetic barrier to resistance in these non-B clades [74]. With increased use of INSTIs by individuals infected with non-B viruses, it is important to monitor these subjects for viral escape in the context of selection pressure [74] and identify the RAM and polymorphic mutations that alter the genetic barrier to resistance and INSTIs efficacy and the role of viral genotype. 

The genetic barrier is measured by evolutionary time to viral escape in the context of a selection pressure [74] and previous studies showed that development of HIV resistance to nucleoside reverse transcriptase inhibitors, non-nucleoside reverse transcriptase inhibitors, and protease inhibitors resulted in part to subtype-associated preferential codon usages and that different amino acid substitutions influenced the likelihood of development of drug resistance [78,79,80]. INSTIs are the newest class of anti-HIV drugs approved for use in HIV/AIDS treatment, and modeling of HIV-1 integrase sequences showed that viral subtypes and codon substitution would differentially affect the genetic barrier to the development of INSTIs resistance [74]. Most mutational pathways linked to higher or lower genetic barrier to resistance to INSTIs come from subtype B infected subjects in resource-rich countries. The role of baseline genetic diversity and subtype-specific polymorphism among non-B subtypes in the development of resistance to INSTIs has not been systematically investigated. With the increasing use of INSTIs worldwide, including in low- and middle-income countries where non-B subtypes predominate, monitoring of nucleotide substitutions that evolves into INSTIs-resistant viruses will help better understand the factors underlying the development of resistance to INSTIs across more diverse HIV subtypes.

Recent studies of patients failing DTG therapy showed that some patients with viral failure had no DRM in the viral integrase but instead had mutations in the nef 3′-PPT region [25,26,27]. These results suggested that mutations outside the integrase, in the 3′-PPT, can confer resistance to DTG and other INSTIs. Furthermore, the 3′-PPT is closely associated with the 5′ terminal of the 3′ LTR, and mutations within the six guanine residues (G-track) of the 3′-PPT can alter RNase H-mediated cleavage at the PPT 3′ terminus, and integrase activity [81,82]. This suggests that mutations in the 3′-PPT G-track might confer an alternative pathway to resistance to DTG and other INSTIs. Thirty-one (9.8%) subjects in our study were infected with viruses that had mutations in the 3′-PPT, including 26 with mutations in the 3′-PPT G-track. The potential effects of these 3′-PPT mutations on integrase function and susceptibility to INSTIs are not known. If DTG/INSTIs-based ART is adopted in Cameroon, studies of patients failing INSTIs-based ART should include both analyses of these 3′-PPT mutations and integrase DRMs to elucidate the potential role of these mutations on the susceptibility to DTG and other INSTIs. 

In summary, the current study of integrase and nef viral sequences from 345 HIV-infected Cameroonians showed only 2 subjects with INSTIs major RAMs, but several subjects with INSTIs accessory RAMs and polymorphic mutations, including 10 subjects harboring viruses that simultaneously had two different INSTIs accessory RAMs. Individually, INSTIs accessory RAMs do not have major effects on susceptibility to INSTIs, but the simultaneous presence of several accessory mutations or their presence in combination with other mutations has been associated with reduced susceptibility to INSTIs, increased viral fitness and virologic failure [40,60,61,62,63,64,65]. With the current worldwide push to expand the use of DTG/INSTIs-based ART, it is inevitable that some patients on these regimens could experience virologic failure at some point during the course of their treatment. Genetic surveillance for management of such cases should include screening for the presence of INSTIs major RAMs, accessory and polymorphic mutations, and potential combinations of these, as well as mutations in the 3′-PPT region. 

## 4. Materials and Methods

### 4.1. Ethics Statement

The study cohort samples were collected as part of an ongoing project aimed at analyzing the influence of HIV genetic diversity on viral neuropathogenesis in Cameroon. This study was conducted in accordance with the Declaration of Helsinki and the protocol was approved by the Cameroon National Ethics Committee (Ethical Clearance Authorization #146/CNE/SE/2012, approved on 13 June 2006 and re-approved on 2 May 2012), as well as the Institutional Review Board of the University of Nebraska Medical Center (UNMC) (IRB #307-06-FB, approved on 26 March 2007 and re-approved annually until 2018). Written informed consent was obtained from all study participants and data were processed using unique identifiers to ensure confidentiality.

### 4.2. Specimen Collection, HIV Serology, CD4 Cell Counts, and Viral Loads

Sample collection, serology, CD4 cell counts, and viral loads analyses were performed in the Hematology laboratory of the Yaoundé University Teaching Hospital or the International Reference Center “Chantal Biya”, Cameroon, between 2008 and 2016. Venous blood samples were collected and stored at room temperature in the outpatient clinic and analyses performed in the Hematology laboratory within 6 h of blood collection. The HIV status of each participant was determined using the rapid immunochromatographic HIV-1/2 test (Abbott Diagnostics, Chicago, IL, USA) and the Murex HIV antigen/antibody Combination ELISA (Abbott Diagnostics), according to the manufacturer’s instructions. A participant was considered HIV positive if he/she tested positive for the two tests, HIV negative if non-reactive for both tests, and discordant if reactive for only one test. No discordant result was observed in our study population.

CD4 T-lymphocyte counts were quantified by flow cytometry, using a Fluorescence-Activated Cell Sorting (FACS) Count Instrumentation System and the BD FACSCount CD4 reagent kit (BD Biosciences, San Jose, CA, USA), according to the manufacturer’s instructions. The FACS instrument was calibrated and quality control tested before each experiment. For viral loads quantification, HIV plasma viral load was quantified by reverse transcription polymerase chain reaction (RT-PCR), using Amplicor HIV-1 Monitor Test (Roche Diagnostic Systems, Pleasanton, CA, USA), according to the manufacturer’s protocol. The assay detection limit was 40 viral RNA copies/mL. For additional molecular analyses, plasma samples were stored in 1mL aliquots at −80 °C until further use.

### 4.3. RNA Extraction and PCR Amplification

Plasma samples were shipped on dry ice (−70 ℃) to UNMC, where sequencing and analyses of the integrase and nef (viral 3′-PPT region) genes were performed. HIV-1 RNA was extracted from plasma using QIAamp Viral RNA mini kit (Qiagen Inc., Germantown, MD, USA) per manufacturer’s protocol. cDNA synthesis and 1st round of PCR amplification of integrase and nef genes was performed using SuperScript^TM^ III One-Step RT-PCR system (Life Technologies, Grand Island, NY, USA). The 50 µL reaction volume contained 500 ng of purified RNA, 25 µL of 2X reaction buffer, 10 pMol of forward and reverse primers (Table 6) and 1 µL reverse transcriptase /Platinum Taq DNA polymerase mix. Reverse transcription was carried out at 50 ℃ for 1 h followed by PCR consisting of an initial denaturation at 94 ℃ for 2 min; followed by 40 cycles of 94 ℃, 15 s; 55 ℃, 30 s; 68 ℃, 1 min; and a final extension step at 72 ℃, 10 min. The nested PCR amplification of integrase and nef genes was carried out in a total volume of 50 µL containing 5 µL of the 1st-round PCR amplicon, 25 µL of 2X KAPA HiFi HotStart ready Mix (KAPA Biosystems, Wilmington, MA, USA), 10 pMol of forward and reverse primers. The nested PCR thermal cycling condition were 95 ℃, 5 min; followed by 35 cycles of 94 ℃, 10s, 60 C, 30s; 72 ℃, 1 min; and a final extension step at 72 ℃, 10 min. Amplicons were detected by agarose gel electrophoresis (1% agarose), visualized by ethidium bromide (0.5 µg/mL) staining under ultraviolet light (260 nm), and images captured using an automated gel documentation system (Syngene, Frederick, MD). Sequences of the primers used for PCR amplifications are detailed in Table 6.

### 4.4. Gene Sequencing 

PCR products were purified using PureLink Quick PCR Purification Kit (Invitrogen, Carlsbad, CA, USA) and subjected to double-strand DNA sequencing to cover the entire amplicon using a set of sequencing primers (Table 6). The sequencing reactions were carried out using the BigDye Terminator v3.1 Cycle Sequencing Kit (Applied Biosystems, Foster City, CA, USA) per manufacturer’s instructions, followed by capillary electrophoresis performed on an Applied Biosystems PRISM 3730 Genetic Analyzer at the UNMC DNA Sequencing Core facility. Sequences of the primers used for DNA sequencing are described in Table 6.

### 4.5. Sequence Analysis of the Study Cohort Samples 

Raw sequence data were manually edited, spliced, and assembled by Sequencher v4.9 to generate the final contig. Multiple sequence alignment of full-length HIV-1 integrase gene was performed with all known HIV-1 group M reference sequences, using Clustal W [85]. All the reference sequences were obtained from the Los Alamos HIV Sequence Database. Phylogenetic trees were constructed using the neighbor-joining method, as well as the Maximum Likelihood method and General Time Reversible model, with 1000 bootstrap replication tests, using MEGA.v.6.0 software [86]. Samples’ HIV subtypes were determined using the NCBI viral genotyping tool. The INSTIs drug resistance associated mutation screening was done using the Stanford University HIV Drug resistance Database v.8.7. [87]. The 3′-PPT and its flanking nucleotide sequences were curated from the full-length nef gene sequences, and their sequence alignments performed using HXB2 as a reference sequence and the Clustal W program [85].

### 4.6. Sequence Analysis of the Database Samples

We analyzed additional Cameroon HIV-1 sequences (integrase and nef/3′-PPT sequences) using full-length HIV-1 sequences from Cameroon previously submitted to the Los Alamos HIV sequence database [28]. We downloaded all Cameroon sequences available in the database and after eliminating duplicate sequences from the same patient, a total of 215 full-length sequences were included in the analysis. Full-length integrase gene sequences for these 215 samples were used to screen for INSTIs RAMs, using the Stanford University HIV Drug resistance Database v.8.7. [87]. The 3′-PPT and its flanking nucleotide sequences were curated from the full length HIV-1 genomic sequences, and their sequence alignments performed using HXB2 as reference sequence and the Clustal W program [85]. 

### 4.7. Statistical Analyses

Comparative analyses of males’ and females’ demographic data were performed using the Student’s *t*-tests (for continuous variables) and Fisher’s exact test (for binary variables). Descriptive statistics including counts and percentages were used to summarize gene polymorphism or mutation occurrences for both cohort and database samples. Fisher’s exact tests were used to compare the proportion of gene polymorphism or mutation occurrences between groups for each gene. False discovery rate (FDR) was controlled to be no more than 0.05 to account for multiple comparisons [88]. All analyses were done using SAS version 9.4 or Prims version 7.0d. 

### 4.8. Data Availability

The full-length HIV-1 integrase and nef gene sequences generated from this study are available in the NCBI database with GenBank accession numbers: MK327828-MK327927 (integrase sequences) and MK333810-MK333910 (nef [including 3′-PPT] sequences).

## Figures and Tables

**Table 1:** Demographic and clinical characteristics of cohort subjects by sex.

Characteristics	Male	Female	*p* Value
N (%)	39 (30%)	91 (70%)	
Age (years; mean ± SD)	37.28 ± 9.94	35.43 ± 8.75	0.29
Age range (years)	18–58	20–56	
Education (years; mean ± SD)	10.50 ± 3.47	9.09 ± 4.03	0.062
CD4 (mean ± SD) (cells /µL)	360.3 ± 320.4	311.2 ± 184.5	0.285
CD4 range (cells /µL)	4–1657	5–894	
Viral load (mean ± SD) (log copies/mL)	4.64 ± 1.54	4.24 ± 1.5	0.174
Viral load range (log copies/mL)	1.60–7.56	1.60–7	
ART-Naïve (N)	33	66	
ART-Experienced (N)	5	25	

N: sample size; SD: standard deviation, ART: antiretroviral therapy.

**Table 2:** INSTIs resistance mutations in cohort samples.

Mutations	HIV-1 Subtype/CRFs							
	A1	CRF01_AE	CRF02_AG	CRF09_cpx	CRF11_cpx	CRF18_cpx	CRF36_cpx	Total (%)
**INSTIs Accessory Resistance Mutations:**								
T97A			1 (1.00)	1 (1.00)				2 (2.00)
E157Q			6 (6.00)					6 (6.00)
**Other Mutations:**								
M50I	2 (2.00)	1 (1.00)	2 (2.00)		1 (1.00)			6 (6.00)
S119R			4 (4.00)					4 (4.00)
L74M			8 (8.00)		1 (1.00)			9 (9.00)
L74I	1 (1.00)		22 (22.00)			1 (1.00)	1 (1.00)	25 (25.00)

INSTIs: integrase strand transfer inhibitors; CRFs: circulating recombinant forms. The number (and percentage) of each mutation are shown for each subtype and CRF.

**Table 3:** Profile of INSTIs drug resistance mutations of database samples.

**Mutation**	**HIV-1 Subtypes/CRFs**										
	**A1**	**A2**	**D**	**F2**	**G**	**K**	**CRF01_AE**	**F2/CRF01_AE**	**CRF02_AG**	**CRF11_cpx**	**CRF13_cpx**
**INSTIs Major Resistance Mutations**											
T66A											
N155K											
**INSTIs Accessory Resistance Mutations**											
T97A	1 (0.47)			1 (0.47)	1 (0.47)			1 (0.47)	5 (2.32)		
A128T											
E157Q			1 (0.47)						1 (0.47)		
**Other Mutations**											
M50I	9 (4.18)	1 (0.47)	1 (0.47)	5 (2.32)	4 (1.86)	1 (0.47)	1 (0.47)	1 (0.47)	5 (2.32)	6 (2.79)	2 (0.93)
L74M	1 (0.47)								6 (2.79)	1 (0.47)	1 (0.47)
L74I	1 (0.47)				1 (0.47)				17 (7.91)		1 (0.47)
S119R					1 (0.47)						
S230N									1 (0.47)		
E138D										1 (0.47)	
**Mutation**	**HIV-1 Subtypes/CRFs**										
	**CRF18_cpx**	**CRF22_01A1**	**CRF36_cpx**	**CRF45_cpx**	**URF**	**M/O recombinant**	**CRF01/A/D/F2**	**N**	**O**	**P**	**Total**
**INSTIs Major Resistance Mutations**											
T66A						1 (0.47)					1 (0.47)
N155K			1 (0.47)								1 (0.47)
**INSTIs Accessory Resistance Mutations**											
T97A					1 (0.47)			1 (0.47)	4 (1.86)	1 (0.47)	16 (7.44)
A128T						1 (0.47)					1 (0.47)
E157Q							1 (0.47)				3 (1.39)
**Other Mutations**											
M50I	1 (0.47)	13 (6.05)		2 (0.93)	2 (0.93)	1 (0.47)		2 (0.93)	10 (4.65)		67 (31.16)
L74M											9 (4.18)
L74I			3 (1.39)	1 (0.47)	1 (0.47)				24 (11.16)		49 (22.79)
S119R											1 (0.47)
S230N	1 (0.47)										2 (0.93)
E138D											1 (0.47)

The number (and percentage) of each mutation are shown for each viral subtype and recombinants.

**Table 4:** Integrase gene mutations in cohort and database AG and non-AG samples.

**Cohort Samples**				
**Gene M**	**AG (*n* = 75)** **(% with M)**	**Non-AG (*n* = 25)** **(% with M)**	***p* Value** **Fisher**	***p* Value** **FDR**
T97A	1.33	4.00	0.43939	0.53221
E157Q	8.00	0.00	0.33214	0.53221
M50I	2.67	16.00	0.03294	0.19764
L74M	10.67	4.00	0.44351	0.53221
L74I	29.33	12.00	0.11097	0.33291
S119R	5.33	0.00	0.56949	0.56949
**Database Samples**				
**Gene M**	**AG (*n* = 61)** **(% with M)**	**Non-AG (*n* =1 19)** **(% with M)**	***p* Value** **Fisher**	***p* Value** **FDR**
T66A	0.00	1.40	0.54941	0.73722
N155K	0.00	1.40	0.54941	0.73722
T97A	17.62	15.36	0.67020	0.73722
A128T	0.00	1.40	0.54941	0.73722
E157Q	3.52	2.79	1.00000	1.00000
M50I	17.62	86.56	0.00000	0.00000
L74M	21.15	4.19	0.00091	0.00501
L74I	59.92	44.67	0.05837	0.21402
S119R	0.00	1.40	0.54941	0.73722
E138D	0.00	1.40	0.54941	0.73722
S230N	3.52	1.40	0.60510	0.73722

M: mutation; FDR: false discovery rate. *p* values based on Fisher exact test and Benjamini–Hochberg FDR are shown.

**Table 5:** Integrase gene polymorphisms in cohort and database AG and non-AG samples.

**Cohort Samples**				
**Gene Polymorphism**	**AG (*n* = 75)** **(% with P)**	**Non-AG (*n* = 25)** **(% with P)**	***p* Value** **Fisher**	***p* Value** **FDR**
E11D	29.33	40.00	0.33308	0.40878
K14R	77.33	56.00	0.06957	0.11049
R20K	9.33	12.00	0.70761	0.73483
A21T	5.33	24.00	0.01449	0.03913
S24N	13.33	24.00	0.21976	0.28255
V31I	68.00	60.00	0.47471	0.53405
L63I	12.00	4.00	0.44389	0.52109
I72V	16.00	44.00	0.00638	0.02462
L101I	89.33	72.00	0.05136	0.08666
T112I	12.00	32.00	0.03120	0.06479
T112V	84.00	64.00	0.04702	0.08666
T124A	90.67	68.00	0.01034	0.03103
T125A	90.67	80.00	0.16850	0.22748
G134D	10.67	28.00	0.05136	0.08666
G134N	90.67	28.00	0.00000	0.00000
I135V	81.33	28.00	0.00000	0.00001
K136Q	0.00	44.00	0.00000	0.00000
K136T	92.00	36.00	0.00000	0.00000
D167E	8.00	28.00	0.01662	0.04079
V201I	96.00	100.00	0.57096	0.61664
T206S	97.33	52.00	0.00000	0.00000
I208L	8.00	20.00	0.13639	0.19381
T218I	28.00	32.00	0.80002	0.80002
L234I	97.33	88.00	0.09808	0.14712
D256E	20.00	48.00	0.00946	0.03103
R269K	30.67	0.00	0.00068	0.00305
S283G	64.00	88.00	0.02489	0.05601
**Database Samples**				
**Gene polymorphism**	**AG (*n* = 61)** **(% with P)**	**Non-AG (*n* = 119)** **(% with P)**	***p* Value** **Fisher**	***p* Value** **FDR**
E11D	26.23	46.10	0.01023	0.01726
K14R	75.41	57.79	0.02262	0.03393
R20K	1.64	5.84	0.26882	0.31558
A21T	8.20	25.97	0.00530	0.01022
S24N	9.84	5.19	0.22585	0.27718
V31I	80.33	50.65	0.00010	0.00024
L63I	9.84	4.55	0.18737	0.24090
I72V	31.15	39.61	0.32761	0.36856
L101I	91.80	55.84	0.00000	0.00000
T112I	9.84	15.58	0.36293	0.39196
T112V	90.16	76.62	0.02780	0.03950
T124A	95.08	78.57	0.00262	0.00545
T125A	95.08	66.23	0.00001	0.00002
G134D	11.48	29.22	0.00863	0.01554
G134N	80.33	39.61	0.00000	0.00000
I135V	75.41	9.09	0.00000	0.00000
K136Q	9.84	51.30	0.00000	0.00000
K136T	85.25	18.83	0.00000	0.00000
D167E	14.75	42.21	0.00020	0.00045
V201I	98.36	89.61	0.06340	0.08559
T206S	91.80	46.10	0.00000	0.00000
I208L	3.28	14.29	0.02228	0.03393
T218I	44.26	12.34	0.00000	0.00002
L234I	98.36	96.10	0.66536	0.69095
D256E	3.28	29.22	0.00001	0.00003
R269K	50.82	5.84	0.00000	0.00000
S283G	70.49	68.83	0.86579	0.86579

P: polymorphism, FDR: false discovery rate. *p* values based on Fisher exact test and Benjamini–Hochberg FDR are shown.

**Table 6:** Primers used for amplification and sequencing of integrase and nef genes [83,84].

Primer ^#^	Position ^^^	Sequence (5′ to 3′)
**Integrase gene 1st PCR**		
IN12	4007 → 4026	GCAGGATTCGGGATTAGAAG
IN13	5270 → 5251	CTTTCTCCTGTATGCAGACC
**Integrase gene 2nd PCR**		
IN1	4137 → 4157	AAGGTCTATCTGGCATGGGTA
BH4	5222 → 5200	TCCCCTAGTGGGATGTGTACTTC
**Nef gene 1st PCR**		
Nef5-1e F1	8513 → 8533	GTGCCTCTTCAGCTACCACCG
Nef3-3e R1	9488 → 9508	AGCATCTGAGGGTTAGCCACT
**Nef gene 2nd PCR**		
Nef5-1e F2	8698 → 8717	TGGACAGAYAGGGTTATAGAA
Nef3-7e R2	9448 → 9468	CACCTCCCCTGGAAAGTCCCC
**Integrase gene sequencing primers**		
IN1	4137 → 4157	AAGGTCTATCTGGCATGGGTA
BH4	5222 → 5200	TCCCCTAGTGGGATGTGTACTTC
IN4764AS	4764 → 4743	CCATTTGTACTGCTGTCTTAA
IN4542S	4542 → 4558	GCAGGAAGATGGCCAGT
**Nef gene sequencing primers**		
Nef5-1e F2	8698 → 8717	TGGACAGAYAGGGTTATAGAA
Nef3-7e R2	9448 → 9468	CACCTCCCCTGGAAAGTCCCC

^#^ (+/+): Sense primer; (+/−): Antisense primer. ^^^ amino acids positions based on the HXB2 numbering system.

## Supplementary Materials

Supplementary materials can be found at https://www.mdpi.com/1422-0067/21/5/1553/s1.

## Abbreviations

HIV	Human immunodeficiency virus
INSTIs	Integrase strand-transfer inhibitors
ART	Antiretroviral therapy
3′-PPT	3′polypurine tract
RAMs	Resistance-associated mutations
CRF	Circulating recombinant form
T	Threonine
A	Alanine
N	Asparagine
K	Lysine
E	Glutamic Acid
Q	Glutamine
M	Methionine
I	Isoleucine
S	Serine
R	Arginine
L	Leucine
D	Aspartic Acid
V	Valine
G	Glycine
C	Cysteine
DNA	Deoxyribonucleic acid
kDa	Kilodalton
Aa	Amino acids
AIDS	Acquired immunodeficiency syndrome
FDA	United States Food and Drug Administration
RAL	Raltegravir
EVG	Elvitegravir
DTG	Dolutegravir
WHO	World Health Organization
SSA	Sub-Saharan Africa
PLWH	People living with HIV
DRMs	Drug-resistance mutations
URF	Unique recombinant form
LTR	Long terminal repeats
UNMC	University of Nebraska Medical Center
ELISA	Enzyme-linked immunosorbent assay
CD4	cluster of differentiation 4
FACS	Fluorescence-Activated Cell Sorting
RT-PCR	Reverse transcription polymerase chain reaction
RNA	Ribonucleic acid
cDNA	complementary DNA
PCR	Polymerase chain reaction
NCBI	National Center for Biotechnology Information
FDR	False discovery rate
