# Supplementary material for: Variability in HIV-1 Integrase Gene and 3′-Polypurine Tract Sequences in Cameroon Clinical Isolates, and Implications for Integrase Inhibitors Efficacy
Source: Int J Mol Sci. 2020 Feb 25;21(5):1553. doi: 10.3390/ijms21051553 (PMC7084836; doi:10.3390/ijms21051553)
Supplement: Supplementary file 1 [file ijms-21-01553-s001.zip › Table S1.pdf]

**Table S1.** Subjects' ID numbers and subtypes of database samples, and the corresponding manuscripts PMID numbers

| SUBJECT ID | PMID | Subtype |
|---|---:|---|
| EU693240 | 18851680 | CRF25_cpx |
| EF087995 | 17725418 | CRF36_cpx |
| EF087994 | 17725418 | CRF36_cpx |
| EF116594 | 17678477 | CRF37_cpx |
| DQ845388 | 17331036 | CRF13_cpx |
| DQ845387 | 17331036 | CRF13_cpx |
| DQ845386 | 17331036 | CRF13_cpx |
| AY169816 | 14678605 | O |
| AY169815 | 14678605 | O |
| AY169813 | 14678605 | O |
| AY169812 | 14678605 | O |
| AY169811 | 14678605 | O |
| AY169810 | 14678605 | O |
| AY169809 | 14678605 | O |
| AY169808 | 14678605 | O |
| AY169807 | 14678605 | O |
| AY169806 | 14678605 | O |
| AY169804 | 14678605 | O |
| AY169803 | 14678605 | O |
| AY169802 | 14678605 | O |
| AJ291718 | 11839159 | CRF11_cpx |
| AY371170 | 15186527 | F2 |
| AY371169 | 15186527 | CRF25_cpx |
| AY371168 | 15186527 | CRF22_01A1 |
| AY371167 | 15186527 | CRF22_01A1 |
| AY371166 | 15186527 | CRF18_cpx |
| AY371165 | 15186527 | CRF22_01A1 |
| AY371164 | 15186527 | A1 |
| AY371163 | 15186527 | CRF22_01A1 |
| AY371162 | 15186527 | H |
| AY371161 | 15186527 | H |
| AY371160 | 15186527 | A1 |
| AY371159 | 15186527 | CRF22_01A1 |
| AY371158 | 15186527 | F2 |
| AY371157 | 15186527 | D |
| AY371156 | 15186527 | D |
| AY371155 | 15186527 | D |

| | | |
|---|---|---|
| AY371154 | 15186527 | CRF13_cpx |
| AY371153 | 15186527 | CRF11_cpx |
| AY371151 | 15186527 | CRF11_cpx |
| AY371150 | 15186527 | CRF11_cpx |
| AY371149 | 15186527 | CRF11_cpx |
| AY371147 | 15186527 | CRF02_AG |
| AY371146 | 15186527 | CRF02_AG |
| AY371145 | 15186527 | CRF02_AG |
| AY371143 | 15186527 | CRF02_AG |
| AY371142 | 15186527 | CRF02_AG |
| AY371141 | 15186527 | CRF02_AG |
| AY371140 | 15186527 | CRF02_AG |
| AY371139 | 15186527 | CRF02_AG |
| AY371138 | 15186527 | CRF02_AG |
| AY371137 | 15186527 | CRF02_AG |
| AY371136 | 15186527 | CRF02_AG |
| AY371134 | 15186527 | CRF02_AG |
| AY371132 | 15186527 | CRF02_AG |
| AY371131 | 15186527 | CRF02_AG |
| AY371130 | 15186527 | CRF02_AG |
| AY371129 | 15186527 | CRF02_AG |
| AY371128 | 15186527 | CRF02_AG |
| AY371127 | 15186527 | CRF02_AG |
| AY371126 | 15186527 | CRF02_AG |
| AY371125 | 15186527 | CRF02_AG |
| AY371124 | 15186527 | CRF02_AG |
| AY371123 | 15186527 | CRF02_AG |
| AY371122 | 15186527 | CRF02_AG |
| AY371121 | 15186527 | G |
| DQ826727 | Unpublished | CRF25_cpx |
| DQ826726 | Unpublished | CRF25_cpx |
| DQ017383 | 16438650 | N |
| DQ017382 | 16438650 | N |
| AY772535 | 15929705 | G |
| AJ271370 | 15199313 | N |
| AJ249239 | 10659053 | K |
| AJ249237 | 10659053 | F2 |
| AJ249236 | 10659053 | F2 |
| AJ239083 | 10438826 | CRF02_AG |
| AJ006022 | 9734396 | N |
| AY623602 | 15321704 | O |

| | | |
|---|---|---|
| AY618998 | 15321704 | O |
| AY532635 | 15320995 | N |
| AY271690 | 12954230 | CRF02_AG |
| AF492624 | 12201907 | CRF11_cpx |
| AF492623 | 12201907 | CRF11_cpx |
| AF460974 | 12201907 | CRF13_cpx |
| AF460972 | 12201907 | CRF13_cpx |
| AF377955 | 11448170 | CRF02_AG |
| AF377954 | 11448170 | CRF02_AG |
| L20571 | 8107219 | O |
| FJ389367 | 19361281 | G |
| FJ389366 | 19361281 | G |
| FJ389365 | 19361281 | G |
| FJ389364 | 19361281 | G |
| FJ389363 | 19361281 | G |
| GQ229529 | 20812894 | CRF22_01A1 |
| AB485667 | Unpublished | O |
| AB485666 | Unpublished | O |
| EU743964 | Unpublished | A1 |
| EU743963 | 20812894 | A1 |
| FN392876 | 20001521 | CRF45_cpx |
| GU201516 | 20426823 | A2 |
| GU201514 | 20426823 | CRF02_AG |
| GU201513 | 20426823 | CRF02_AG |
| GU201512 | 20426823 | CRF02_AG |
| GU201511 | 20426823 | CRF02_AG |
| GU201508 | 20426823 | A1 |
| GU201505 | 20426823 | F2/CRF01_AE recombinant |
| GU201504 | 20426823 | CRF02_AG |
| GU201500 | 20426823 | CRF02_AG |
| GU201499 | 20426823 | CRF02_AG |
| GU201498 | 20426823 | CRF02_AG |
| GU201497 | 20426823 | CRF02_AG |
| GU201495 | 20426823 | CRF02_AG |
| GU201494 | 20426823 | CRF02_AG |
| GQ324962 | 20059396 | N |
| GQ324959 | 20059396 | N |
| GQ324958 | 20059396 | N |
| GU237072 | 20518650 | J |
| HQ179987 | 21084486 | P |
| JN864059 | 22549382 | A1 |

| | | |
|---|---|---|
| JN864057 | 22549382 | CRF02_AG |
| JN864055 | 22549382 | CRF02_AG |
| JN864053 | 22549382 | CRF02_AG |
| JN864051 | 22549382 | CRF22_01A1 |
| JN864049 | 22549382 | CRF22_01A1 |
| JN864047 | 22549382 | A1 |
| JN864058 | 22549382 | A1 |
| JN864056 | 22549382 | G |
| JN864052 | 22549382 | CRF02_AG |
| JN864050 | 22549382 | A1 |
| JN864048 | 22549382 | A1 |
| JX140673 | Unpublished | F2 |
| JX140647 | Unpublished | CRF02_AG |
| JX140676 | Unpublished | G |
| JX140672 | Unpublished | F2 |
| JX140670 | Unpublished | D |
| JX140646 | Unpublished | CRF02_AG |
| KF716465 | Unpublished | CRF22_01A1 |
| KF716464 | Unpublished | CRF02_AG |
| KF716463 | Unpublished | CRF22_01A1 |
| KF716462 | Unpublished | CRF22_01A1 |
| KF716461 | Unpublished | CRF22_01A1 |
| KF716460 | Unpublished | CRF22_01A1 |
| KF859740 | Unpublished | CRF02_AG |
| KF859739 | Unpublished | CRF02_AG |
| KP109503 | Unpublished | CRF11_cpx |
| KP109502 | Unpublished | G |
| KP109501 | Unpublished | D |
| KP109500 | Unpublished | CRF22_01A1 |
| KP109499 | Unpublished | CRF22_01A1 |
| KP109498 | Unpublished | CRF22_01A1 |
| KR822830 | Unpublished | CRF11_cpx |
| KP718938 | Unpublished | CRF11_cpx |
| KP718937 | Unpublished | CRF11_cpx |
| KP718936 | Unpublished | CRF11_cpx |
| KP718935 | Unpublished | CRF11_cpx |
| KP718934 | Unpublished | CRF11_cpx |
| KP718933 | Unpublished | URF |
| KP718932 | Unpublished | URF |
| KP718931 | Unpublished | CRF18_cpx |
| KP718930 | Unpublished | CRF01_AE |

| | | |
|---|---|---|
| KP718929 | Unpublished | CRF11_cpx |
| KP718928 | Unpublished | A1 |
| KP718927 | Unpublished | URF |
| KP718926 | Unpublished | CRF13_cpx |
| KP718925 | Unpublished | G |
| KP718924 | Unpublished | CRF13_cpx |
| KP718923 | Unpublished | G |
| KP718922 | Unpublished | CRF02_AG |
| KP718921 | Unpublished | URF |
| KP718920 | Unpublished | CRF11_cpx |
| KP718919 | Unpublished | CRF45_cpx |
| KP718918 | Unpublished | A1 |
| KP718917 | Unpublished | CRF37_cpx |
| KP718916 | Unpublished | D |
| KP718915 | Unpublished | G |
| KP718914 | Unpublished | CRF11_cpx |
| KR017779 | 26354000 | F2 |
| KR017778 | 26354000 | CRF11_cpx |
| KR017777 | 26354000 | CRF02_AG |
| KR017776 | 26354000 | G |
| KR017774 | 26354000 | CRF36_cpx |
| KR017773 | 26354000 | CRF02_AG |
| KR017772 | 26354000 | URF |
| KR017771 | 26354000 | CRF02_AG |
| KU168295 | 26699702 | O |
| KU168294 | 26699702 | O |
| KU168293 | 26699702 | O |
| KU168285 | 26699702 | O |
| KU168281 | 26699702 | O |
| KU168265 | 26699702 | CRF02_AG |
| KU168311 | 26699702 | A1 |
| KU168310 | 26699702 | CRF02_AG |
| KU168307 | 26699702 | CRF02_AG |
| KU168306 | 26699702 | G |
| KU168305 | 26699702 | A1 |
| KU168304 | 26699702 | CRF02_AG |
| KU168303 | 26699702 | CRF02_AG |
| KU168302 | 26699702 | G |
| KX228824 | Unpublished | CRF02_AG |
| KX228823 | Unpublished | CRF02_AG |

| | | |
|---|---|---|
| KX228819 | Unpublished | A1 |
| KX228817 | Unpublished | CRF22_01A1 |
| KX228816 | Unpublished | CRF22_01A1 |
| KX228809 | Unpublished | F2 |
| KX398187 | Unpublished | CRF02_AG |
| KX579838 | Unpublished | O |
| KU749422 | Unpublished | F2 |
| KU749421 | Unpublished | CRF02_AG |
| KU749420 | Unpublished | F2 |
| KU749419 | Unpublished | CRF02_AG |
| KY498771 | 28193549 | N |
| KY658700 | Unpublished | CRF11_cpx |
| KM438032 | Unpublished | M/O Recombinant |
| KM438031 | Unpublished | M/O Recombinant |
| MF767262 | 29575910 | N |

PMID: Unique identifier number used in PubMed
